# Endosulfan induces reproductive & genotoxic effect in male and female Swiss albino mice

**DOI:** 10.1186/s42826-024-00208-4

**Published:** 2024-05-22

**Authors:** Priya, Arun Kumar, Mohammad Ali, Abhinav Srivastava, Ranjit Kumar, Ashok Kumar Ghosh

**Affiliations:** 1 Mahavir Cancer Sansthan and Research Centre, Patna, Bihar 801505 India; 2 Nagaland University, Zhenuboto, Nagaland India

**Keywords:** Endosulfan, Free radical assay, Graffian follicles, Estrogen hormone, Testosterone hormone, Chromosomal aberrations, Histopathology

## Abstract

**Background:**

Toxicity by pesticide has become a global health issue and leaves a harmful impact on human health via various ways. The people exposed to pesticides in the rural population get affected by the harmful effects of it as they enter the human body system through skin, inhalation, oral administration, food chain and many more ways. The present work is designed to study the toxic effect of endosulfan in male (n=30) and female (n=30) Swiss albino mice. Endosulfan was administered by oral gavage (oral administration) method, at the dose of 3.5 mg/Kg body weight daily for period of 3 weeks, 5 weeks and 7 weeks. After the completion of the treatment, the mice were sacrificed and their ovary and testis tissues were dissected out to check the degeneration. The blood was collected for karyotyping, biochemical and hormonal analysis of pesticide induced genotoxicity. After 7 weeks of administration with Endosulfan, various abnormalities were observed in male and female mice.

**Results:**

Treatment with endosulfan at the dose of 3.5 mg/Kg body weight caused a higher degree of degeneration in the reproductive organ of Swiss albino mice . Treatment by this pesticide generated degeneration in long duration of dosage for 3,5 and 7 weeks. Ovaries of endosulfan administered groups showed degenerated germinal epithelium, Graffian follicles and corpus luteum. In testis of endosulfan treated mice, microscopic examination showed that there is significant damage and reduction in the tissue of seminiferous tubules and primordial germ cells. High degree of degeneration caused the disarrangement and deformation of spermatogonia with the decrease in the number of Sertoli cells. Biochemical and hormonal properties was also affected by endosulfan treatment. There was significant 5 folds decrease in the testosterone value of endosulfan in 7 weeks treated mice in comparison to control (p < 0.0001) and similarly there was significant elevation in the estrogen levels found in 7th week endosulfan treated mice. It also influenced the level of free radicals as there was significant decrease (p < 0.0001) in the value in catalase levels in 7 weeks endosulfan treated male and female mice, while significant (p < 0.0001) increase in the values of lipid peroxidation levels as 8 folds and 10 folds in 7 weeks endosulfan treated male and female Swiss albino mice respectively. This study hence speculates that the endosulfan exposed population are at the risk of reproductive health hazards.

**Conclusions:**

The present study thus concludes that, endosulfan after 7 weeks of exposure caused significant reproductive damage to both male and female Swiss albino mice groups. Moreover, the karyotyping study also correlated the genotoxic damage in the mice.

## Background

The toxicity of pesticides has emerged as a major concern for public health on a worldwide scale and has several negative effects on human health. Endosulfan is an organochlorine insecticide that is a member of the cyclodiene chemical family. Its chemical name is hexachloride-hexahydro-methanol-benzodioxathiepinoxide. According to a research [1], it is a cyclic sulphurous acid ester with the chemical formula C_9_H_6_O_3_Cl_6_S and the molecular weight of 407 g/mole. The term “pesticide” refers to a substance that is often employed in agriculture or for the protection of public health and is made up of a combination of several chemicals. In order to demonstrate protection for plants against pests, weeds, and illnesses, as well as protection for people against diseases that are transmitted by vectors, such as malaria, dengue fever, and schistosomiasis [2, 3]. According to study [4], such examples include insecticides, fungicides, herbicides, rodenticides, and plant growth regulators. According to previous data [5], the use of agrochemicals and pesticides, in general, has become an essential component of agricultural systems around the globe over the course of the last century. This has made it possible for a discernible rise in crop yields and the production of food. Pesticides, herbicides, rodenticides, bactericides, fungicides, and larvicides are the several types of pesticides that may be classified according to their uses and the organisms that they are designed to kill. Organophosphate is the most widely used pesticide, followed by organochlorine, pyrethroids, and carbonates. When compared to other pesticides, carbonates are the least effective. Herbicide amide is the most widely used herbicide, followed by phenoxy hormone products, bipyridyl, triazines, urea derivatives, diniooanilines, carbarnate herbicide, and sulfosulfuron. Sulfosulfuron is the least used herbicide. Herbicide is the most widely used kind of pesticide, followed by fungicide and bactericide, then insecticide, and finally plant growth regulators [6]. According to a report [7], the usage of endosulfan, which was intended to benefit humankind, has instead had the unintended consequence of making the ecosystem more vulnerable. Both and endosulfan are components of the pesticide known as endosulfan, which is employed in agricultural production of crops [8, 9]. Rare cases of acute or chronic endosulfan exposure have been documented, however it is possible for purposeful intake to cause serious health problems or even death [10]. According to the findings of investigations on the acute and chronic toxicity of endosulfan in animals, the primary organs that are affected are the kidneys, the liver, the immune system, and the reproductive system [11]. According to a study conducted by a researcher [12], India is the second biggest producer of pesticides in Asia, behind only China. On a worldwide scale, India ranks 12th and accounts for around 2% of the entire market. Endosulfan has the ability to cause changes in the cholinergic, dopaminergic, and serotonergic actions that occur in the central nervous system. Accordingly, there has been a significant amount of interest produced in the investigation of a link between endosulfan-induced behavioural aberrations and alterations in the activities of neurotransmitters [13]. Because of its widespread use and the possibility that it may be transported by the environment, this pesticide has been shown to be contaminating the sample of water, soil, the environment as a result of evaporation, and agricultural products [14]. When rats were subjected to a combination of endosulfan for varying amounts of time, alterations were seen in the tissue of their livers, kidneys, and muscles. These findings allowed for the histopathological aspect to be comprehended. According to a report [15], endosulfan has effects that are necrotic to the muscles, nephrotoxic to the kidneys, and hepatotoxic. One of the most significant contributors to the development of acute leukaemia is being exposed to pesticides. Child leukaemia, which is one of the most serious illnesses, has been observed in a study after the intake of a pesticide [16]. These studies also reveal that absorption of endosulfan contamination may occur through a variety of pathways and for a variety of reasons, resulting in a variety of disorders. The goal of this research is to determine how endosulfan affects the reproductive capabilities of Swiss Albino mice. Furthermore, the research investigates the genotoxic impact of Endosulfan after 7 weeks of exposure.

## Results

### Biochemical analysis - level of catalases in different experimental group

In different experiment shows decrease (p < 0.0001) in the value of catalase in endosulfan treated group as compared to control group of mice. Significant decrease (*p* < 0.0001) in the treated group of catalases have been reported in male and female Swiss albino mice groups (Tables 1 and 2).

**Table 1:** Level of catalases (male) in different experimental groups

Week	Control	Treated	p-value
3rd week	1.70 ± 0.26	0.97 ± 0.08	< 0.0001
5th week	1.59 ± 0.23	0.42 ± 0.12	< 0.0001
7th week	1.62 ± 0.23	0.32 ± 0.13	< 0.0001
- Value	NS	0.0001	

Level of catalases in male mice (values are expressed in ± SD, *p* < 0.005)

**Table 2:** Level of catalases (female) in different experimental groups

Week	Control	Treated	p- Value
3rd week	1.99 ± 0.20	1.07 ± 0.40	< 0.0001
5th week	1.90 ± 0.29	0.70 ± 0.22	< 0.0001
7th week	1.94 ± 0.52	0.50 ± 0.28	< 0.0001
p- Value	NS	0.0001	

Level of Catalases in female mice (Values are expressed in ± SD, *p* < 0.005)

### Biochemical analysis - level of lipid peroxidation (MDA) in different experimental group

In different group of an experiment shows increase in the value of Lipid in endosulfan treated group as compared to control group of mice. Significant increase in the treated group of catalases have been reported in male and female Swiss albino mice groups (Tables 3 and 4).

**Table 3:** Level of lipid peroxidation (MDA) (Male) in different experimental group

Week	Control	Treated	p- Value
3rd week	0.42 ± 0.06	1.68 ± 0.18	< 0.0001
5th week	0.51 ± 0.07	2.77 ± 0.80	< 0.0001
7th week	0.46 ± 0.07	3.98 ± 0.83	< 0.0001
p- Value	NS	0.0001	

Level of LPO in male mice (Values are expressed in ± SD, *p* < 0.005)

**Table 4:** Level of lipid peroxidation (MDA) (Female) in different experimental group

Week	Control	Treated	p-Value
3rd week	0.44 ± 0.05	1.83 ± 0.56	< 0.0001
5th week	0.45 ± 0.10	2.63 ± 0.65	< 0.0001
7th week	0.44 ± 0.11	4.55 ± 1.38	< 0.0001
p- Value	NS	0.0001	

Level of LPO in female mice (Values are expressed in ± SD, *p* < 0.005)

### Hormonal analysis

The serum testosterone level showed decreased levels after endosulfan exposure in comparison to the control (Table 5).

**Table 5:** Serum testosterone levels in male mice in different experimental group

Week	Control	Treated	p- Value
3rd week	0.46 ± 0.14	0.28 ± 0.08	< 0.0001
5th week	0.46 ± 0.12	0.28 ± 0.10	< 0.0001
7th week	0.43 ± 0.09	0.23 ± 0.31	< 0.0001
p- Value	NS	0.0001	

Level of serum testosterone in male mice (Values are expressed in ± SD, *p* < 0.005)

In this study female mouse indicates estrogen levels elevated in endosulfan administered group after longer period of treatment that is 3 weeks, 5 weeks, and 7 weeks (Table 6).

**Table 6:** Serum estrogen levels in female male mice in different experimental group

Week	Control	Treated	p-Value
3rd week	0.20 ± 0.06	0.31 ± 0.07	< 0.0001
5th week	0.22 ± 0.05	0.35 ± 0.10	< 0.0001
7th week p- Value	0.21 ± 0.06 NS	0.27 ± 0.09 0.0001	< 0.0001

Level of serum estrogen levels in female mice (Values are expressed in ± SD, *p* < 0.005)

### Histopathological study of the testis

**Fig. 1:**
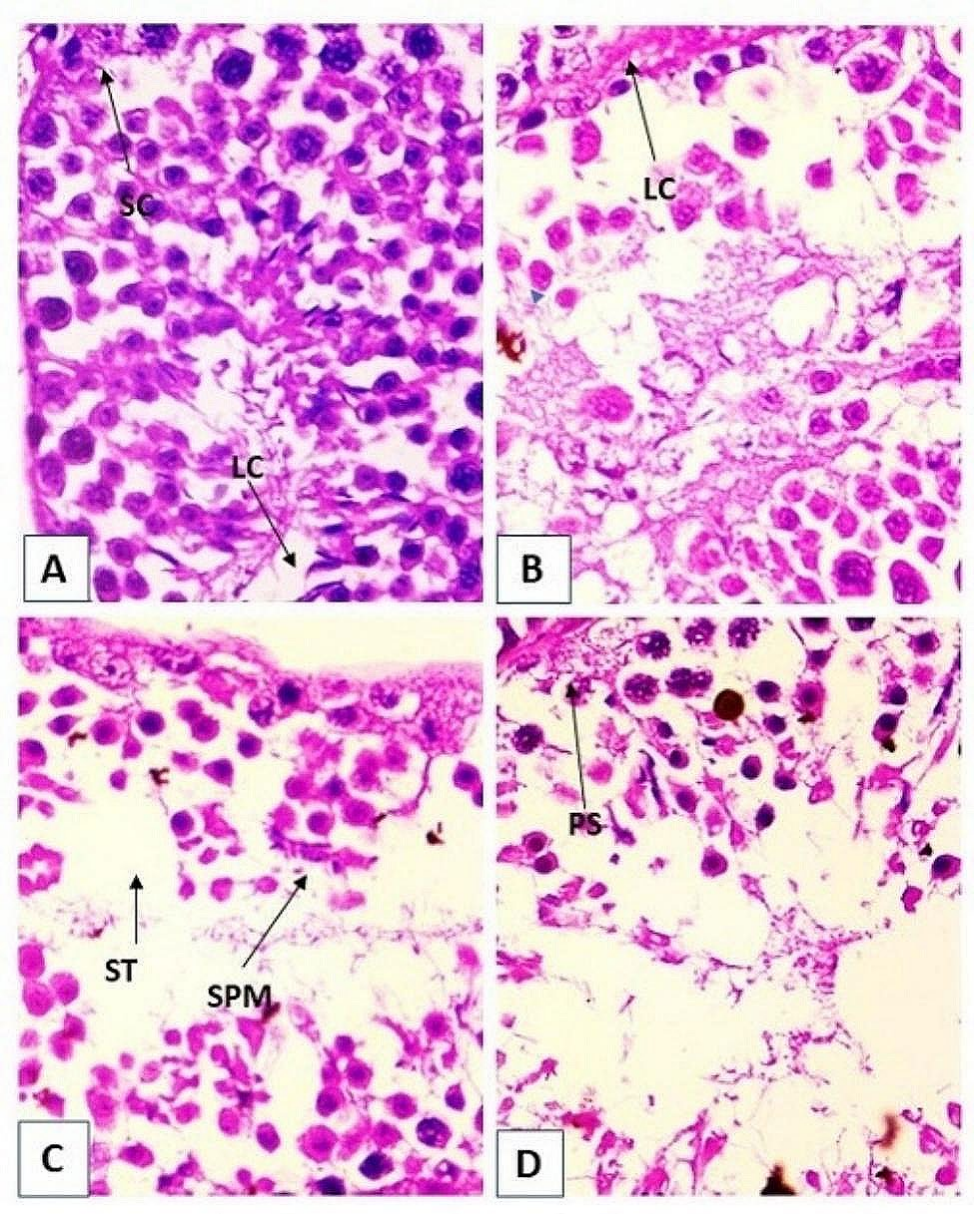
Microphotograph of mice testis-stained with haematoxylin and eosin (H&E) x400. (**A**) Section of control mice testis tissue showing normal arrangement of spermatocyte (SC). (**B**) Testis tissue section of 3rd week endosulfan treated group mice showing tubulated and degenerated form. (**C**) Testis tissue section of 5th week endosulfan treated mice testis showing altered distribution and development of seminiferous tubules (ST) and spermatogonia (SPM). (**D**) 5 weeks endosulfan treated mice testis showing altered distribution of and shape of seminiferous tubule and primary spermatocyte (PS)

In the present study, there was significant degeneration observed in the seminiferous tubule of the endosulfan treated mice group in comparison to the control. The section showed normal histoarchitecture of spermatogenetic cycle as the primary spermatocyte, spermatogonia, spermatids and spermatozoa were well arranged in the seminiferous tubule (Fig. 1A). There was gradual degeneration in the primary spermatocytes and spermatogonia with vacuolation in the seminiferous tubules after endosulfan treatment for 3 weeks, 5 weeks and 7 weeks respectively (Fig. 1: A, B,C & D).

### Histopathological study of the ovary

**Fig. 2:**
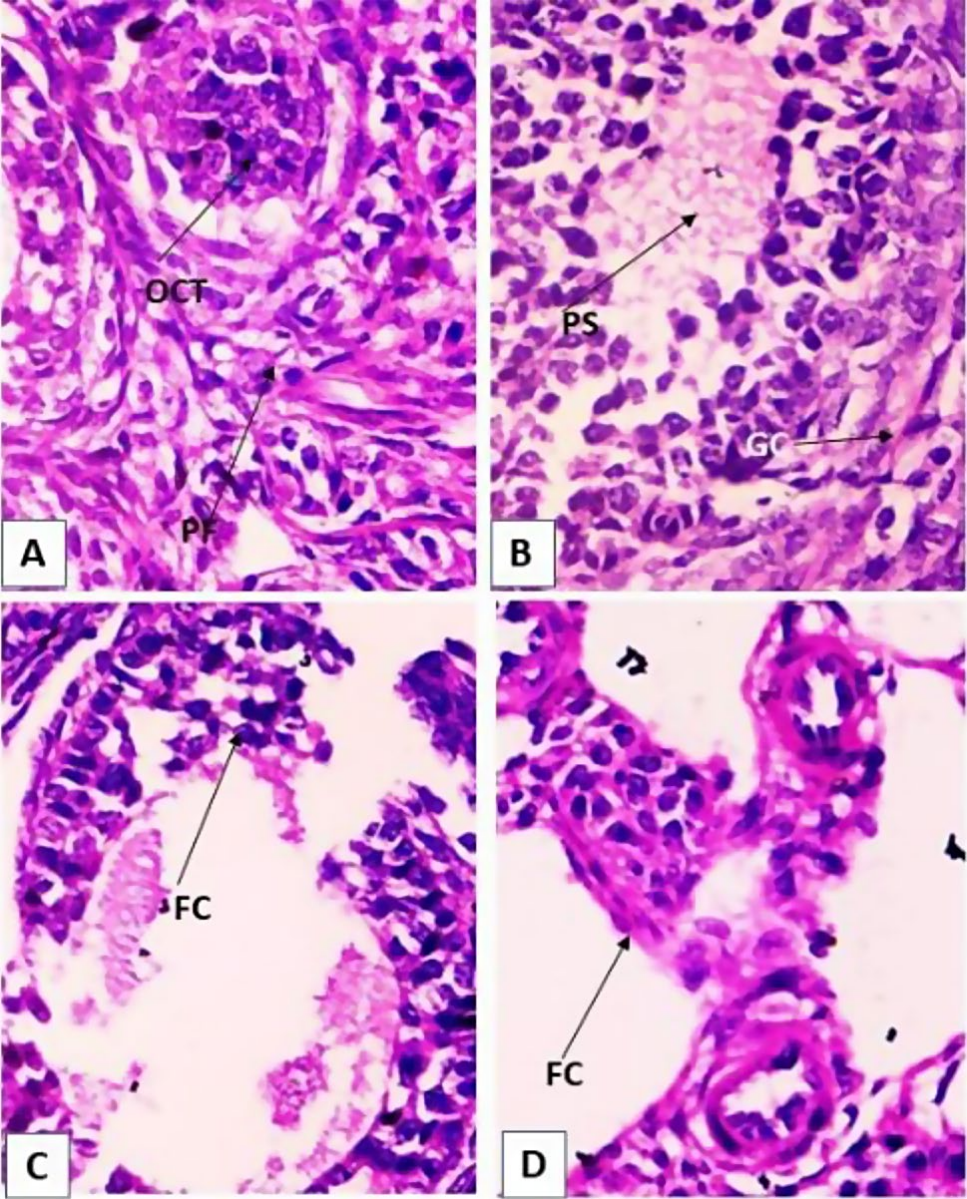
Microphotograph of mice ovary-stained with haematoxylin and eosin (H&E). x 400. (**A**) Ovary of control mice showing normal and mature Graffian follicle with completely normal Oocyte (OCT). (**B**) Ovary of 3rd week endosulfan administered mice showing many vacuolated spaces were observed in matured Graffian follicles with clustered nuclei of granulosa cells and 1˚ spermatocyte. Degeneration in ova was also prominent. (**C**) Ovary of five weeks endosulfan administered mice showing degenerated germinal cell. Many vacuolated spaces were observed in matured Graffian follicles (GF) with clustered nuclei of granulosa cells (GC). Degeneration in ova was also prominent. (**D**) 7 weeks endosulfan treated ovary showing degenerated germinal epithelium. Many vacuolated spaces were observed in matured Graffian follicles with clustered nuclei of Granulosa cells

The present study also showed that endosulfan administered mice caused degeneration of germinal epithelium to a greater extent. Corpus luteum was observed in highly degenerated condition along with germinal epithelium was also fragmented. Large vacuolated spaces and abnormally distributed with Graffian follicles were observed in mature with different primordial follicles. Degeneration in ova were observed with clustered nuclei of granulosa cells. Serrated chromosome arm with degeneration in centromere were prominently observed (Fig. 2A, B, C & D).

### Chromosomal abnormalities

**Fig. 3:**
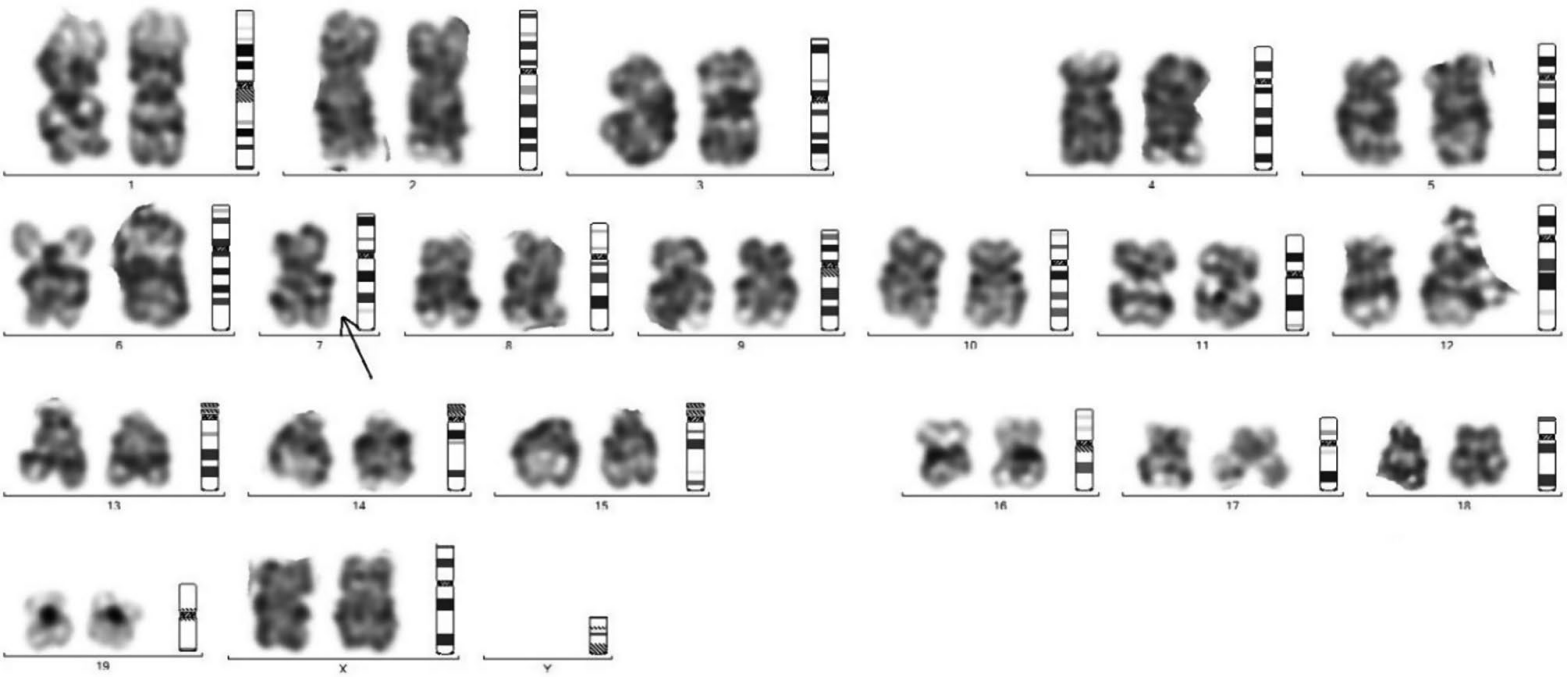
Chromosomal study of male untreated control mice: Showing normal centromere and long arm and short arm in normal condition. Chromosome of female control mice showing normal centromere and long arm and short arm in normal condition

**Fig. 4:**
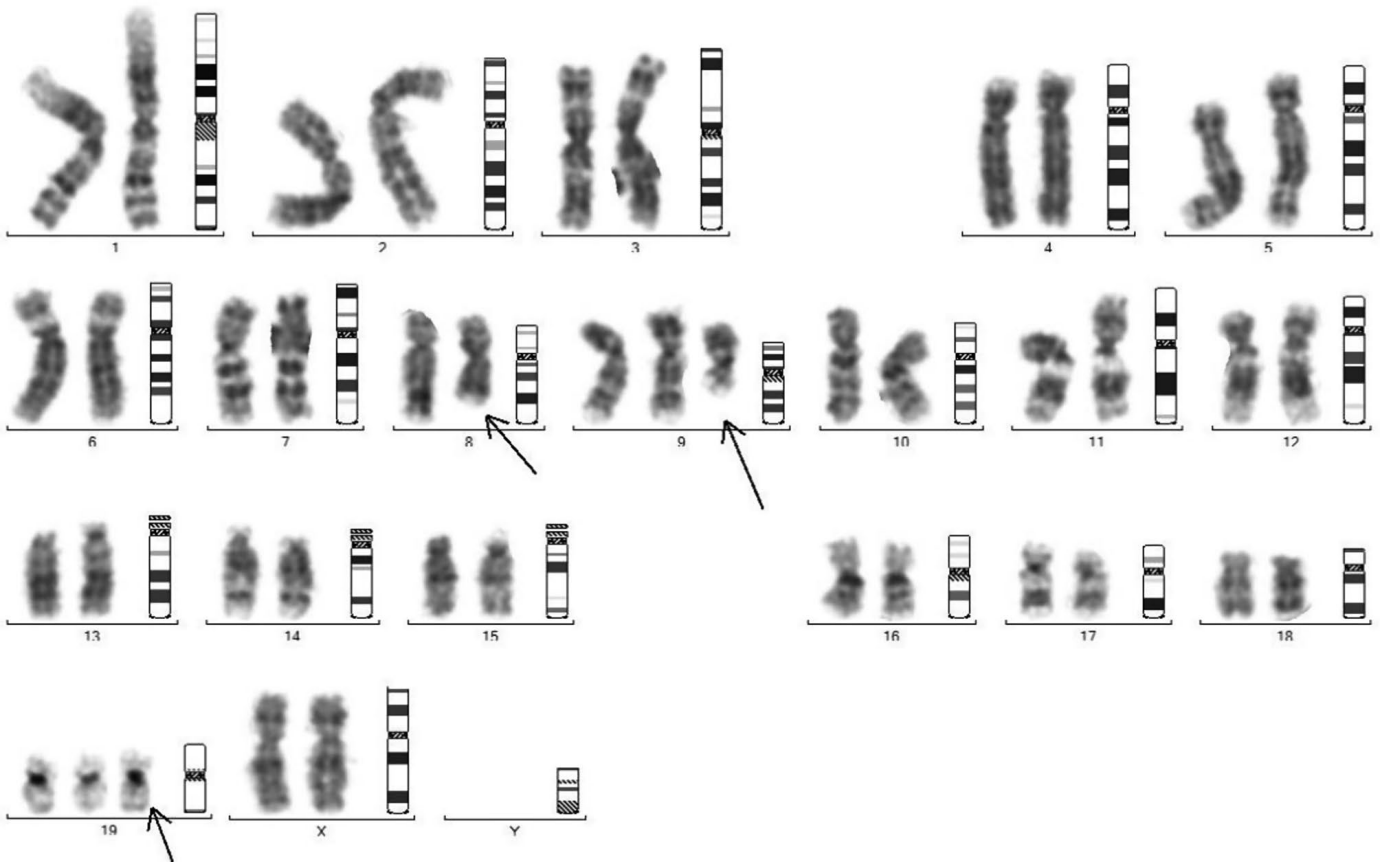
Chromosomal study of Endosulfan 3 weeks treated mice: Showing slight loop formation in arms, Chromosome of endosulfan treated mice shows elongation in both arms of chromosome

**Fig. 5:**
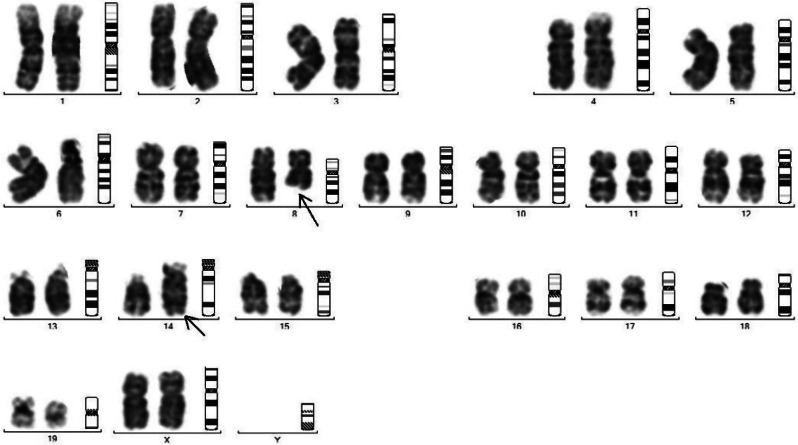
Chromosomal study of Endosulfan5 weeks treated mice: Showing irregular shape and bulging in arms Chromosome of treated mice shows irregular shape and elongation in chromosome in both arms

**Fig. 6:**
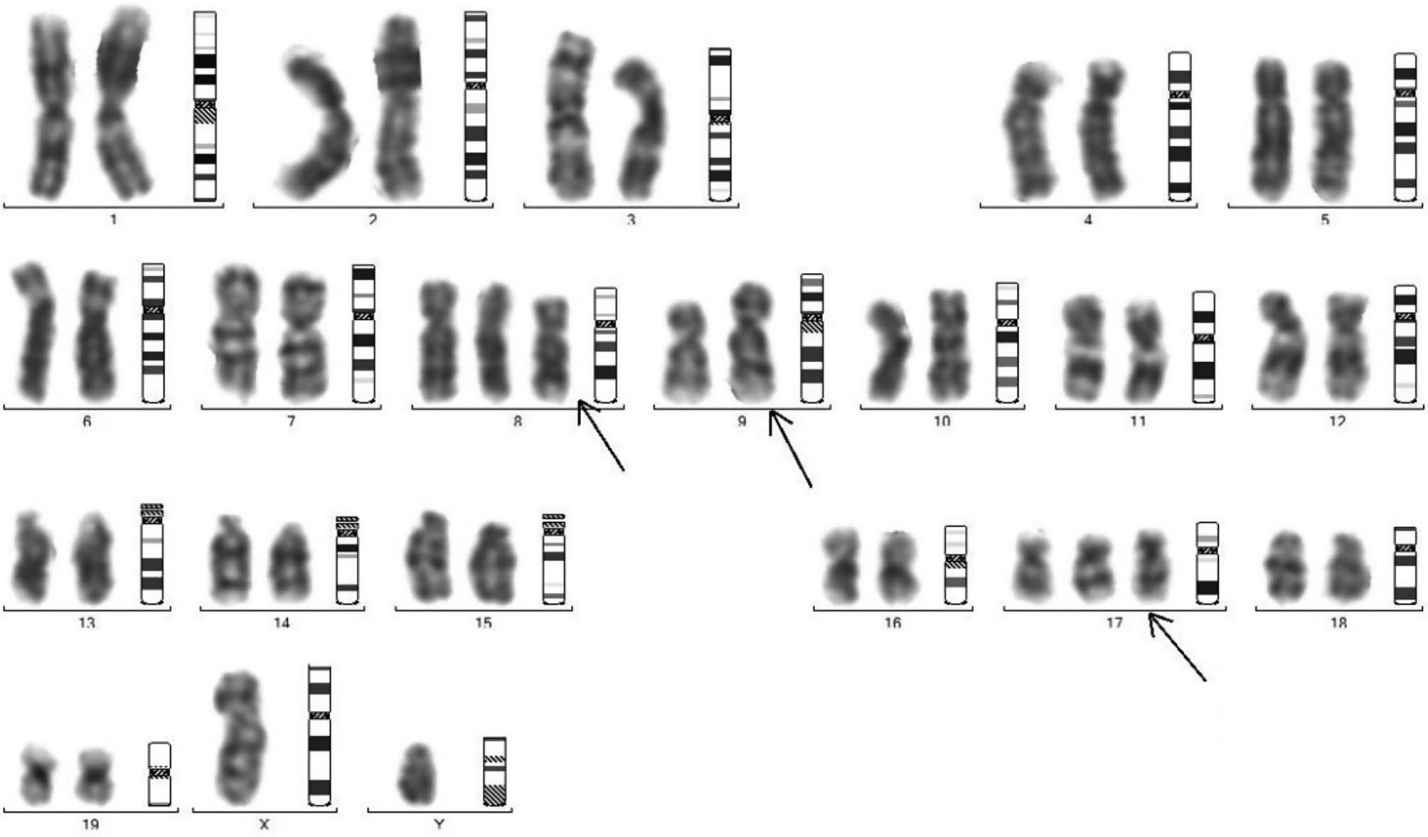
Chromosomal study of Endosulfan 7 weeks treated mice: Showing elongation in both arms of chromosome and also shows irregular shape, bulging and elongation in arms of chromosome

Fragmented chromosomal arms with little dense telomeres were also observed indicating early loss of the genes residing at telomere end and responsible for cellular stress including ageing (Figs. 3, 4, 5 and 6).

## Discussion

Endosulfan is a xenoestrogen pesticide causes serious damage to the vital organs of the body. In the present study, it has been observed that endosulfan caused deleterious effect on the reproductive tissues of Swiss albino mice especially the ovaries in female and testis in males. The findings showed remarkable histological degeneration in the Graffian follicles, granulosa and germinal epithelium in 3 weeks, 5 weeks and 7 weeks endosulfan treated mice groups. Moreover, similar degeneration was observed in the male mice especially in primary spermatocytes, spermatogonia, spermatids and spermatozoa in seminiferous tubules. The most fascinating findings were the chromosomal aberrations in both male and female mice groups. The hormonal findings also showed significant decrease in the testosterone levels while significant increase in the estrogen levels respectively. The present study also identifies, the magnitude of the endosulfan toxicity with increase in the dose durations. Furthermore, the most significant finding observed was the chromosomal aberrations in 7 weeks endosulfan treated group, which signifies and correlates with the hormonal and biochemical results. Moreover, the magnitude of the degeneration was very much high in these groups.

Some researchers [17, 18] found that endosulfan caused testicular damage in animals and the impact of it on human reproductive health is particularly significant in the southern region of India, particularly in the state of Kerala. Instead of having a direct harmful effect on the organ, it has a significant impact on the hormones’ ability to do their work. Individuals are affected by this pesticide because it lowers down the quality of their sperm and their sperm counts [19, 20]. An investigation of the impact that prenatal exposure to endosulfan has on the process of spermatogenesis in male children who have reached puberty was carried out in the context of this research. This phase of exposure is especially significant since day 12 of gestation, when fetal gonads begin to differentiate [21], as a result, this period of exposure is particularly crucial. The pesticide causes tissue damage at the organismal level, and its impact has been seen particularly in the testes, where it leads to the mortality of testicular cells and reducing the number of testicular cells. Endosulfan exposure causes alterations in the testes that are reliant on spermatogenesis [22]. These changes result in a decrease in sperm counts and motility, which leads to male infertility. According to a study endosulfan causes cytotoxicity in Sertoli–germ cell co-cultures, which causes disruption in the normally occurring connection between Sertoli and germ cells, which in turn leads to malfunction in the testicles [23]. The treatment of endosulfan on the testis had a detrimental effect on the level of tissue in its deteriorated condition. Male mice that were treated with endosulfan showed signs of degraded spermatid and secondary spermatocyte, both of which induce an azoospermic state and leads to infertility [24]. According to the research done by a group of researcher the testosterone propionate has the potential to counteract the effects of endosulfan on the immunological adherence function of erythrocytes [25]. According to a probable pathogenetic mechanism of the toxicity connected to apoptotic activity in the testis cells ultimately results in testicular degeneration and has an effect on spermatogenesis [26]. Endosulfan exposure at the crucial period of time, when gender determination occurs may alter the histoarchitecture of the testis as well as the balance between the proliferation and apoptosis of testicular cells [27]. The researchers conducted research by administering a similar chemical to female mice and discovered that after three weeks, there was only a moderate degree of degeneration. However, after five and seven weeks, increasingly significant degrees of degeneration were seen. After 56 days of treatment with endosulfan, it was noticed frequent vacuolization in ova in addition to degeneration of Graffian follicles, granulosa cells, and the germinal epithelial layer [28]. According to the findings of the research, endosulfan is responsible for the decrease in testosterone levels that mice undergo. According to a report one of the factors that contributes to infertility is the degeneration of nuclear material that has many vacuolated gaps [29]. The levels of free radicals were significantly affected by the induction of this pesticide, which occurred in a dose-dependent way [30]. Oxidative stress was the consequence of free radicals, which played a key part in the development of testicular injury and low sperm counts. Endosulfan plays a significant part in the alterations that take place in the hormonal levels of male and female mice in a variety of distinct ways, and it also has an impact on the body weight, which discovered that the testosterone level in male mice was lower than the amount seen in the control group [31]. . It has been observed that the increased levels of testosterone hydroxylation in the endosulfan were related with an overall rise in the rate of androgen excretion from the mice that was 2–3 times higher than before. This enhanced excretion may have brought about a little but insignificant decrease in the amounts of testosterone seen in the blood. According to a study the synthesis of testosterone is affected by feedback regulatory regulation [32]. It was postulated in a study that the effect of this pesticide causes alterations in hormonal pattern due to its estrogenic nature [33]. Following treatment with endosulfan, a decline in both the testosterone level and the sperm counts was noted. A group of researchers find that, there was a significant reduction in motility as well as sperm count, both of which contributed to male infertility by impairing reproductive health. According to study the combination of the pesticide and endosulfan has a very harmful impact on the levels of testosterone in male rats [35, 36]. Endosulfan, when given to female Swiss albino mice for a month, significantly exacerbated the uterine abnormalities that were already present [37]. Micrographs of testes from normal mice indicated a substantial difference between control and treated animals in research that evaluated the effect of a pesticide [38]. The disruption of Leydig cells and seminiferous epithelia was followed by a fall in testosterone levels [39]. In contrast to male mice, it had a detrimental effect on female mice, and this effect manifested itself as an increase in the amount of estrogen. Endosulfan, in its most basic form, acts in a manner similar to that of estrogen in the case of female Swiss albino mice [40]. According to research when a pregnant woman or nursing mother comes into contact with a pesticide, the possibility exists for the foetus and the nursing kid to be exposed to endosulfan in a variety of different ways [41]. In a trial that lasted for six weeks and included producing endosulfan in mice, the study found that pesticide exposure led to increased estrogen levels in the animals. There was a considerable decline in the number of corpus luteum, which led to changes in the mice’s estrogen production. According to study infertility in mice occurs as a result of the degeneration of the corpus luteum, which results from a lack of the process that negatively affects ovulation [42]. In a study conducted on lymphocyte, the genotoxic impact of endosulfan was observed with undesirable effect on gene level by inducing DNA damage. Furthermore, it was also found that the toxic impact of endosulfan on several aspects of reproductive activities in male and female Swiss albino mice is highly unique and substantial, and it correlates all the analysed parameters. This was discovered as a result of the findings of the research that was described above [43, 44]. In addition to this, there was significant damage at the chromosomal level, which is indicative of the aberrant functioning in the gonadal tissues that contribute to sterility in those tissues. Because of this, endosulfan is a genotoxic substance that poses significant risks to the health of human population who are exposed. As a result of this, the findings of the current research suggests that farmers should immediately stop using this pesticide.

### Conclusions

The present study thus, concludes that endosulfan adversely affects histological parameters in male and female reproductive system in dose dependent manner along with the long-term exposure by endosulfan in male and female mice results in fragmentation, elongation and bulging of the chromosome in both arms. It also influences the hormonal value by decreasing the level of testosterone and increasing the level of estrogen. There were also significant biochemical changes in lipid peroxidation and catalases levels in treated groups. It also influences the level of free radicals in long term treatment. This study hence recommends that, the endosulfan exposed population are at the risk of reproductive health hazards therefore the use of this pesticide should be discontinued for agricultural purposes.

## Methods

### Pesticide

Endosulfan (CAS No- 115-29-7; Product No. 32,015; Batch No. BCBT1374) procured from Sigma Aldrich (Buchs, Switzerland) was used as pesticide for toxicity induction. The calculated dose of effective concentration − 3.5 mg/Kg body weight for the Swiss albino mice was used after the estimation of LD_50_ value.

### Experimental animals

Male (n = 30) and female (n = 30) Swiss albino mice were provided by the animal house of Mahavir Cancer Sansthan and Research Centre, Patna, India. Diet including food and water to mice were given (prepared by laboratory itself). Mice were segregated 5 weeks before the start of the experiment in male and female different groups and were acclimatized. These experimental mice were housed in standard polypropylene cages having 2 animals in each cage and were randomly distributed into control and treatment groups. The temperature of the animal house was maintained at (24 °C ± 2 °C) for the rats with 12-hour light/dark cycle. The age group of mice selected for the study was 12 weeks old with 30 ± 2 gm body weight. The animal had free access of water and food *ad libidum.*

### Experimental model for toxicity assessment

The experimental animals were grouped in the following cages. Group-I: Control, Group-II: E- 3 weeks, Group-III: E- 5 weeks, Group IV: E-7 weeks. The endosulfan was treated to the group II – IV with the dose of 3.5 mg/Kg body weight per day.

### Experimental design

The endosulfan at the dose of 3.5 mg/Kg body weight per day was treated to the group II – IV for 3 weeks, 5 weeks and 7 weeks respectively. After the completion of the entire experiment, the animals were sacrificed. The mice were anesthetized with mild diethyl ether before the dissection. The blood and the tissues from the control and the treated mice were collected for the study. Blood samples of mice of each group were taken in separate small test tubes by orbital sinus puncture. After separation of the serum, it was collected in separate small, labelled vials and were kept in refrigerator for the biochemical analysis. Ovaries and testis from the sacrificed mice were removed and washed three times in isotonic saline (0.85 gm/100 mL) and fixed for the subsequent histological studies under light microscopy. For the histopathological analysis mice were sacrificed from each group. Ovaries and testis were dissected out and washed three times in isotonic saline (0.85 w/v %) and then fixed in neutral formalin solution for histopathological study.

### Biochemical analysis

Serum was collected for hormonal analysis and biochemical assays- lipid peroxidation (MDA) and Catalases.

### Lipid peroxidation assay

Thiobarbituric acid reactive substances (TBARS), as a marker of LPO, were evaluated through the double heating based on the principle of spectrophotometric measurement of colour reproduced during the reaction to thiobarbituric acid (TBA) with malondialdehyde (MDA). For this study, 2.5 mL of 10% solution of trichloroacetic acid (TCA) was mixed with 0.5 mL serum in a centrifuge tube and heated in the water bath for 15 min. After cooling at room temperature, the mixture was further allowed to centrifuge at 3000 rpm for 10 min, and 2 mL supernatant was mixed with 1mL of 0.675% TBA solution in a test tube which was further heated in water bath at 90 °C for 15 min and left for cooling at the room temperature. Thereafter, further absorbance was measured by UV-visible spectrophotometer (UV-10, Thermo Scientific) at 532 nm.

### Catalase assay

The blank, control and sample in 3 different test tubes were taken followed by addition of phosphate buffer and distilled water 1 mL and 0.4 mL respectively. The H_2_O_2_ were added to blank and sample after addition of distilled water followed by addition of 2 mL of potassium dichromate. Finally kept in hot water bath for 15 min at boiling temperature followed by cooling at room temperature and analysis by UV spectrophotometer at 570 nm.

### Hormonal assay

Hormonal assessment was done using the enzyme-linked immunosorbent assay (ELISA) method, and Estrogen Immunotag Mouse T Elisa Kit (Lot No. 202,711) and Testosterone Immunotag Mouse T Elisa kit (Lot No. 202,711) were measured by a kit manufactured by Biosciences. (Page Avenue, St. Louis, MO, USA).

### Histopathological study

In the histopathological study, to study the changes in testis and ovaries tissues of various groups of mice, permanent mounts of the testis and ovaries tissues were prepared after the mice were sacrificed, testis and ovarian tissues were dissected out and washed through the normal saline and fixed in 10% formalin for 24 h. The tissues were then dehydrated through graded series of ethanol and then were embedded into paraffin. The section of 5 μm was cut through digital microtome (Thermo Fisher Digital microtome HM340E) and were stained with Haematoxylin and Eosin (H&E) for histopathological investigation under light microscope.

### Karyotyping study

For the blood culture and chromosomal analysis, heparinized whole blood was cultured for 72 h in RPMI 1640 supplemented with 20% Fetal calf serum, 2% phytohemagglutinin and antibiotics (100 IU/mL penicillin and 100 Lj.g/mL streptomycin). Two hours before harvesting 0.2 ig/ml Colchicine was added to the culture. Prepared slides were stained with Giemsa stain and 100 metaphases were screened for chromosomal aberrations such as breaks, exchange, dicentrics, ring and acendric fragments. Whole chromosomal sets were final analysed.

### Statistical analysis

Data comparisons are carried out by using one way analysis of variance (ANOVA) followed by Turkey’s multiple range test (TMET). Results were presented as mean ± SD along with the statistical significance among the studied groups for various parameter such as biochemical, LPO and hormonal assay are analysed by using software Graph Pad Prism programme (Graph Pad Software, Inc., San Diego, USA). Differences were considered significant if *p* < 0.05.

## Data Availability

The datasets used and/or analysed during the current study are available from the corresponding author on reasonable request.

## Abbreviations

*GC*: Granulosa cells
*GF*: Graffian follicles
*OCT*: Oocyte
*PS*: Primary spermatocyte
*SC*: Spermatocyte
*SPM*: Spermatogonia
*ST*: Seminiferous tubules
*LAB ANIMAL*: Laboratory Animal Research
